# An Original Approach to Evaluating the Quality of Blood Donor Selection: Checking Donor Questionnaires and Analyzing Donor Deferral Rate

**DOI:** 10.3389/fmed.2018.00074

**Published:** 2018-03-21

**Authors:** Philippe Gillet, Esther Neijens

**Affiliations:** ^1^Service du Sang, Belgian Red-Cross, Suarlée, Belgium

**Keywords:** blood donor selection, transfusion safety, evaluation, donor health questionnaire, donor deferral rate

## Abstract

Blood donor selection is a cornerstone for blood transfusion safety, designed to safeguard the health of both donors and recipients. In the Service du Sang, Belgian Red Cross, French and German-speaking part of Belgium (SFS), health professionals (HPs) are allowed to interview donors on their own after formal qualification. This qualification is afterward evaluated by means of two complementary quality indicators: monitoring of donor health questionnaires (DHQs) and analysis of donor deferral rate. The study aims to evaluate the degree to which both quality indicators may be useful and appropriate tools to evaluate the quality of blood donor selection. An analysis performed on 2016 data showed that noncompliance detected by means of DHQ monitoring seems to be more frequent in HPs who conduct a low number of interviews compared to all HPs as a group (5.67 vs. 3.23%; *p* < 0.001). Deferral rates are also higher in HPs with a lower activity compared to HPs who interview more donors (14.80 vs. 13.00%, *p* < 0.001). Furthermore, statistically differences are observed between the type of blood donation venue in terms of the global deferral rate (for instance fixed site vs. schools: 11.9 vs. 19.5%; *p* < 0.001), and specific reasons for deferral (such as sexual risk behavior and travel in at-risk areas, the differences being highly significant between each category of blood donation venue; *p* < 0.001). Providing the HPs with feedback on these findings was an opportunity to draw their attention to some aspects of the selection process in order to improve it.

## Introduction

Blood donor selection is a cornerstone for blood transfusion safety, designed to safeguard the health of both donors and recipients. Donor safety (1, 2) is targeted by reducing the risk of complications associated with blood donation (rare but not absent) (3) and, in order to improve recipient safety, blood donor selection attempts to reduce the risk of transfusion-transmitted infections (TTI). A recent study conducted in Senegal confirmed what is globally accepted based on epidemiological data, i.e., the efficacy of blood donor selection in reducing the prevalence of HIV seropositive donors (4).

The European Directive 2005/62/EC (5) recommends entrusting the responsibility for blood donor selection to qualified health professionals (HPs). Training in blood donor selection encompasses both a theoretical and a practical approach: the HP has to be able to take the right decision (knowledge of blood donor selection criteria) and to communicate this decision to donors in an appropriately understanding manner (professional relationship with each donor based on trust).

The blood donor selection process usually encompasses four main steps (6):

- Pre-donation information and advice: this is usually provided in a leaflet, especially about transfusion-transmitted infections (and the associated risk factors) and the potential risks of donation.
- Donor Health Questionnaire (DHQ): filled out by the donor alone (before the pre-donation interview) or with the HP (during the pre-donation interview).
- Donor interview: conducted by a qualified HP.
- Donor health assessment: at the end of the interview, the donor is declared eligible to give blood or deferred temporarily or permanently; this decision also takes into account physical and biological parameters, such as hemoglobin level, blood pressure, heart rate, and weight.

Before an HP is allowed to conduct donor interviews and selection alone, qualification must be acquired.

In the Service du Sang, Belgian Red Cross, French and German-speaking part of Belgium (SFS), the following qualification criteria are used and as recommended in terms of training objectives (7), communicated to the HPs at the start of the training session:

- Theoretical training is provided (possibly *via* e-learning) focusing on blood donor selection criteria: a predefined grade must be achieved in order to continue the training,
- the ability to take the right decision is assessed in simulated cases by a senior HP/supervisor,
- a certain number of donor interviews is overseen by a senior HP/supervisor in order to assess the trainee’s ability to take the right decision and to communicate properly with donor, especially when the donor is deferred,
- the supervisor monitors the first interviews performed alone by checking the donor health questionnaires (DHQs) (see below).

Each step must be successfully completed before the next step is taken. When all steps are completed, the HP is qualified for donor selection.

After this initial qualification, HPs are allowed to interview donors alone, but their qualification is not infinite. It is nevertheless difficult to evaluate the quality of this activity. As confidentiality is a prerequisite for the pre-donation interview, regular attendance by a third person (supervisor) is not recommended even with a view to evaluating the competency of each HP. Furthermore, the presence of a third person could influence the behavior of both the donor, who might hesitate to disclose all the relevant data [donors’ compliance is not always achieved (8, 9)] and the HP, who might be more inclined to apply all the procedures than when conducting the interviews alone.

Blood donor selection by qualified HPs can be evaluated in various ways. For example, the quality of the decision taken by a trainee (nurse, technician, or physician) can be assessed by comparison with the decision taken by a senior physician, the latter being considered as the reference (10). In the SFS, the qualification of each HP is formally monitored through two main quality indicators, the monitoring of DHQs and the donor deferral rate:

- A sample of DHQs filled in by donors and analyzed by the HPs during the pre-donation interview are monitored yearly for each HP in order to assess whether the HP in question collected the relevant data and used them properly.
- The donor deferral rate observed for each individual HP is compared to the overall rate for all HPs within the SFS in order to detect differences that might reflect different ways of selecting donors.

Both quality indicators are used routinely by the SFS. The study aims to analyze their adequacy to evaluate the quality of blood donor selection and to possibly improve blood transfusion safety.

Results of both indicators are provided to each individual HP to improve their blood donor selection, and, as a consequence, to improve blood transfusion safety.

Evaluations performed on blood donor selections, which took place in 2016 in the SFS are analyzed and the results reported in the study that provides some examples of issues detected and thoughts about the further development of these tools.

## Materials and Methods

The study was conducted as an audit of blood donor medical selection analyzing two indicators: DHQs monitoring and blood donor deferral rate. This audit was submitted to the Erasme-ULB Ethics Committee (Brussels, Belgium), which determined that full review and approval was not required.

### DHQs Monitoring

In the SFS, each HP ideally undergoes one annual monitoring by a senior HP (supervisor). The paper version of the DHQ filled in by the donor and checked by the HP with the donor during the face-to-face interview, the data entry in the data-base (eProgesa^®^ software, MAK systems, Paris, France) and the decision taken are checked for a series of DHQs, typically all the DHQs for one blood collection session for this HP.

The DHQ used by the SFS is made up of 43 questions, of which 42 are to be answered by ticking a yes/no box. One text answer is mandatory, i.e., country of birth. The DHQ must mandatorily be dated and signed by both the donor and the HP. In addition, HPs are asked to document the interview and their reading and checking of the DHQ with the donor by writing a comment and/or additional info and/or “ok” or at least by adding a checkmark next to the answer provided by the donor, if this answer could be a potential reason for deferral or when the “yes” answer generates a specific laboratory analysis request when entered into the data-base. This is the case for example for the question “Have you ever had a malaria attack?” which induces the detection of malaria antibodies.

### Blood Donor Deferral Rate

Data collected in eProgesa from January to December 2016 involving all donations and including HPs identification, decision taken by the HP (donor acceptance or deferral), reason for deferral (when the donor is deferred), and type of blood donation venue (fixed site or mobile site, i.e., village, school, office, or special) were analyzed.

### Statistical Analysis

In order to evaluate HPs’ compliance in donor selection, the following items are checked, according to the DHQ used by the SFS. These items are listed in a standardized questionnaire control form (QCF) used by supervisors in order to document the control:

- Donor signature
- HP signature
- Donor’s “Yes” answers documented
- Answers to all questions
- Coherent answer on DHQ and entry into data-base
- Donor selection.

Data from all QCFs collected in 2016 were recorded in an Excel data-base and analyzed by descriptive statistics (mean, median, and percentiles), including the identification of HPs, the blood donation venue, the category of donor (first-time vs. regular or repeat donor), and the six types of noncompliance listed above.

Blood donor deferral rates were calculated globally and for each specific deferral category for the whole group of HPs, for HPs who conducted fewer than 500 interviews during the research period and for those who conducted 500 interviews or more. Deferral rates in the various blood donation venues were compared. The statistical analysis was performed by using the chi square test in order to evaluate the degree to which observed differences were statistically significant.

## Results

During the study period, 209,617 pre-donation interviews were conducted by 125 HPs (Table 1).

**Table 1:** Activity of health professionals (HPs).

2016	SFS	Fewer than 500 pre-donation interviews	500 or more pre-donation interviews
HP	125	34	91
Pre-donation interviews	209,617	10,861	198,756
Interview/HP	1,677	319	2,184

Out of these 125 HPs, 57 (46%) were assessed with a total of 2,063 DHQs. More HPs may have been monitored but were not included in the study because the QCFs were not provided on time.

Deferral rates were calculated and analyzed for all donations.

### Donor Health Questionnaires

This study reports data from 2,063 DHQ checks carried out within the SFS, i.e., 0.98% of all DHQs for 2016.

Table 2 provides data analyzed from the 57 monitored HPs. The proportion of DHQs assessed for first-time donors is 12.4% (256 out of 2,063).

**Table 2:** Monitoring of donor health questionnaires.

	No. of health professionals (HPs)	No. of questionnaires	No. of first-time donors	Questionnaires not OK	Donor’s signature missing	HP’s signature missing	Response not documented	No response to at least one question	Discrepancy between questionnaire and decision	Quality of decision
SFS	57	2,063	256	117	5	57	22	62	10	19
%				5.67%	0.24%	2.76%	1.07%	3.01%	0.48%	0.92%
Median				0.00%	0.00%	0.00%	0.00%	0.00%	0.00%	0.00%
P10				0.00%	0.00%	0.00%	0.00%	0.00%	0.00%	0.00%
P75				4.76%	0.00%	2.94%	0.00%	1.69%	0.00%	0.00%
P90				8.09%	0.00%	5.91%	4.46%	4.86%	0.00%	3.02%

*Data were analyzed from 57 HPs whose questionnaires were monitored in 2016*.

Noncompliances for the various parameters checked by means of the QCF, as described above, are displayed in absolute numbers and in percentages of the total number of DHQs checked for all HPs as a group. The distribution according to median and P10, 75 and 90 shows great inter-individual variability.

Table 3 provides data analyzed from a total of 1,610 DHQs from HPs who interviewed more than 500 donors during the study period (*N* = 47), i.e., a selection of HPs who performed donor selection most regularly.

**Table 3:** Monitoring of donor health questionnaires (more than 500 pre-donation interviews).

	No. of health professionals (HPs)	No. of questionnaires	No. of first-time donors	Questionnaires not OK	Donor’s signature missing	HPs signature missing	Response not documented	No response to at least one question	Discrepancy between questionnaire and decision	Quality of decision
SFS	47	1,610	193	52	3	34	14	22	2	9
%				3.23%	0.19%	2.11%	0.87%	1.37%	0.12%	0.56%
Median				0.00%	0.00%	0.00%	0.00%	0.00%	0.00%	0.00%
P10				0.00%	0.00%	0.00%	0.00%	0.00%	0.00%	0.00%
P75				4.27%	0.00%	1.14%	0.00%	0.00%	0.00%	0.00%
P90				8.09%	0.00%	5.08%	3.02%	4.63%	0.00%	2.56%

*Data were analyzed from HPs who have interviewed more than 500 donors during the study period*.

For the whole group of HPs (Table 2), 5.67% of the DHQs were rejected by the supervisor.

The most frequent noncompliance issue was a missing answer, i.e., no box ticked by the donor and no comment provided by the HCP: 3.01% of the checked DHQs.

The second most frequent noncompliance was missing HP signature (2.76%).

The least frequently reported noncompliance was missing donor signature: 5 times out of 2,063, i.e., 0.24%.

Overall, the quality of the decision (donor acceptance or deferral) was considered as being wrong in 0.92% of the cases.

The HP subgroup who interviewed most donors on an annual basis (more than 500) had lower percentages of noncompliance than the whole group for all parameters checked (Table 3).

A great variability was observed for the various criteria in both the whole group of HPs and in the group of HPs who interviewed more than 500 donors during the study period.

### Blood Donor Deferral Rate

A total of 13.09% of the 209,617 donors interviewed by the 125 HPs were deferred for blood donation (Table 4).

**Table 4:** Comparison of deferral rates between health professionals (HPs) who conducted fewer than or at least 500 pre-donation interviews during 2016.

2016		SFS (%)	Fewer than 500 pre-donation interviews (%)	500 or more pre-donation interviews (%)	*p*
Deferral rate	μ	13.09	14.80	13.00	<0.001
	σ	5.95	9.84	3.28	
Recording rate	μ	85.12	78.47	85.54	<0.001
	σ	12.72	14.33	11.50	
Sexual risk behavior deferral rate	μ	1.09	1.25	1.08	NS
	σ	0.68	0.80	0.64	
Travel deferral rate	μ	1.57	1.25	1.59	<0.01
	σ	1.01	0.89	1.05	
Exposure to potential TTI	μ	1.60	1.58	1.60	NS
	σ	0.56	0.73	0.48	

*TTI, transfusion-transmitted infections; NS, not significant*.

The deferral reason was recorded by the HPs in the data-base during the pre-donation interview for 85.12% of donors.

The main reasons were:

- Exposure to potential transfusion-transmitted infectious agents within the deferral period, due to surgery, endoscopy, tattoo, piercing, potentially contaminating accident, acupuncture, mesotherapy, or blood transfusion: 1.60% (traveling and sexual risk behavior are not included in this item).
- Traveling in a region where transfusion-transmitted infectious agents are present, mainly malaria, Chagas disease, and West Nile Virus infection: 1.57%.
- Sexual risk behavior, i.e., new sexual partner within the deferral period, men having sex with men, or sexual partner infected with HIV, HBV, or HCV: 1.09%.

Other deferral reasons, such as current infection or serious medical condition were less frequent.

Quite a number of donors (4.5%) were not allowed to give blood, but blood analyses were performed in order to check previous results. The main reason is probably low hemoglobin level, but this information is not available for analysis.

Table 4 provides data on deferral rates for the group of HPs as a whole, for HPs who interviewed fewer than 500 donors and for HPs who interviewed at least 500 donors during the same study period. SDs suggest major inter-individual differences.

In HPs who interview more donors (at least 500/year) overall deferral rates were lower and, more interestingly, variability tended to decrease, particularly for the global deferral rate (SD: 9.84% compared to 3.28%).

Furthermore, statistically significant differences are observed between the type of blood donation venue, i.e., fixed or the various types of mobile sites (Table 5). For instance, there is a significant difference in global deferral rates (Table 5) between fixed and mobile sites, such as schools, offices, and special campaigns (for instance, during periods requiring a call to potential donors by media). Differences between each category (fixed sites, villages, schools, offices, and special drives) are highly significant with respect to sexual risk behavior and travel in at-risk areas (Table 5).

**Table 5:** Comparison of donors’ deferral rate between fixed and mobile sites.

2016	Fixed sites (%)	Villages (%)	Schools (%)	Offices (%)	Special (%)	Comments
Overall deferral rate	11.9	12.3	19.5	16.0	19.5	No difference between schools and special drives *p* < 0.05 between fixed sites and villages *p* < 0.001 between all other categories
Sexual risk behavior	0.92	0.66	4.03	1.39	2.32	*p* < 0.001 between all categories
Travel	1.40	0.98	1.79	3.91	2.82	*p* < 0.001 between all categories
Exposure to potential transfusion-transmitted infections	1.19	1.70	2.78	1.83	2.82	*p* < 0.001 between all categories (except between villages and offices, and between schools and special: not significant)

Data for each individual HP are compared with those of the group as a whole and to those of HPs working in the same type of venue as the monitored HPs. This comparison allowed to identify differences that were discussed with the HP, an opportunity to assess their blood donor selection activity and, when needed, to provide additional training.

## Discussion

The analysis of the data suggests that checking DHQs and analyzing blood donor deferral rate on an individual basis may be used as quality indicators of blood donor selection and as a basis for improvement of this activity.

### Donor Health Questionnaires

The completion of DHQs is a useful tool to provide HPs with adequate data to take the right decision. Using a DHQ reduces the risk of transmission of blood-borne infectious agents by transfusion. A study showed a significant reduction in Gabonese seropositive donors (hepatitis B, hepatitis C, and syphilis) who completed a DHQ (11), although this encouraging finding was not observed in all studies (12), probably partly because of differences in the prevalence of pathogens.

One limitation of the DHQ is its understanding by all donors; its form has to be regularly analyzed and reviewed (13, 14), in particular, to ensure donors’ compliance with respect to questions about sexual risk behaviors (15, 16); nevertheless, direct questions pinpointing sexual behavior may reduce donor’s intention to return for a further donation (especially in the case of experienced blood donors) (16).

Another limitation of the DHQ is the noncompliance on the part of some donors when it comes to responding properly and honestly to all questions. To improve donors satisfaction and probably to make donors more compliant when it comes to filling out the DHQ honestly, an abbreviated questionnaire may be an alternative at least for repeat and regular donors (17).

A standardized DHQ is used by most blood services to collect relevant data in order to evaluate the donor’s eligibility to give blood. Each blood service uses its own standardized DHQ. Exceptions are the USA and Canada, where uniform DHQs have been used since 2005. In Germany, a national DHQ has been recently tested (16).

An automated computer-assisted pre-donation interview could improve the collection of relevant data (14, 18) but in most countries, the pre-donation interview is conducted without the assistance of a computer and remains a human activity. An evaluation of this activity could, therefore, be useful to improve donor selection.

These critical findings highlight the importance of analyzing DHQs as a means to evaluate the quality of the blood donor selection, confidentiality required by the donor interview conflicting with the attendance of a supervisor.

In this study, monitoring DHQs by means of the QCF allowed to identify a number of issues which otherwise would have gone unnoticed and adapted feedback was provided to the HPs concerned.

Some issues occured only once, as for any human activity: one HP for example, forgot to make sure that an answer was given to one question in the set of DHQs checked. This HP was then reminded of the importance of staying focused. If several questions remained unanswered, the HP received different feedback and was possibly monitored for a certain period until they improved.

An issue such as a missing HP signature is major, but was mostly due to simple forgetfulness on the part of the HP who made an adequate donor selection. In fact, as HPs are asked to comment/sign off on all “yes” answers that might be a reason for deferral, tracking their comments made it possible to confirm whether the DHQ has been checked during donor selection. Missing signatures could, therefore, be noticed by the supervisor in most cases.

The same type of noncompliance issues detected during the DHQ checks may have a very different impact on transfusion safety. The impact may be possible transmission of a TTI, a health consequence for the donor, or rather noncompliance with the legal framework which may have no immediate consequences for safety in a specific case, but should of course be avoided.

Therefore, it is important that the supervisor who performs the checks have thorough knowledge of the consequences of all issues, and give appropriate feedback to the HP involved.

The supervisor adapts their evaluation of the HP when performing DHQ controls accordingly. The examples below illustrate this individualized approach:

- A not documented “yes” answer or no answer to the question:
  When the question involves antecedents of allergy or asthma, the consequence is rather minor, whereas when it concerns contact with a person suffering from hepatitis or another infectious disease, this may have major consequences. In fact, the HP may have missed a reason for deferral or for requesting additional laboratory analyses, with potential transmission of a TTI.
- Discrepancies between DHQ completion and data entry:
  For instance, it is more important to enter the actual “yes” answer in the data-base for the question “Have you ever had a malaria attack?” in the case of a first-time donor than for “Have you ever been operated on since you were born?” Indeed, a positive answer to antecedents of malaria automatically generates an analysis request and possibly deferral of the donor. Therefore, if the appropriate answer is not entered, the opportunity for an analysis request and/or donor deferral will be missed.
- Donor selection:
  Missing deferral of a donor who is at risk of having been infected with a blood-borne agent, such as a donor who has a new sexual partner within the deferral period, can have a safety impact. On the other hand, accepting a donor whose hemoglobin level is 0.1 g/dL below the legal threshold for giving blood is wrong, but has no safety consequences for either the recipient or the donor.

The least frequent noncompliance issue observed in this study, the missing donor signature, is a major one as without this confirmation from the donor, the blood bag cannot be used.

Sometimes systematic or very frequent issues were detected in some or all of the HPs in a group, or in an individual HP. For the supervisor, this was an opportunity to remind the HPs of the rules to be followed in order to improve blood collection quality and safety. In some cases, the issue was a subject for a continued education session.

Examples:

- The majority of HPs in a group understood the question involving a “donor born in a malaria region, who has lived in this region for the 5 first years of their life?” as living in a malaria region for the *full* first 5 years. Therefore, if the donor has only lived in this region for 1 year, the answer entered was “no,” resulting in the malaria analysis request not being automatically generated on the first blood donation. Not testing for malaria could miss a reason for deferral and transmission of malaria. How to answer this question correctly was explained to the HP group twice by the supervisor, first by email when it became obvious that this was a recurrent error, and then included in the next continued education session. After these two steps, there was a marked improvement.
- One HP did not check properly in which countries Chagas disease is present and forgot to record the relevant journey. As a result, the analysis request was not generated.

The reminder to this HP to check whether a country is affected by Chagas disease had an obvious impact on their data entry.

In some situations, incorrect documentation has no impact on blood quality or safety, but can give the wrong impression of noncompliance. This may induce the presence of findings during an audit or inspection.

For instance, one HP always entered the date of the interview as the start date for a temporary contra-indication (e.g., following a tattoo) instead of the actual date; the end date, however, was correctly calculated. The donor was thus deferred for the correct duration and there was no safety issue whatsoever. However, a *post hoc* administrative check would identify the duration of the deferral as being too short and consider it to be a failure to comply with legal requirements. The HP was thus reminded to enter the actual start date and subsequent checks confirmed that this was being done correctly.

Noncompliance seems to be more frequent in HPs who conduct fewer interviews (5.67 vs. 3.23%; *p* < 0.001). This information is interesting and should be analyzed further in order to assess whether there is a threshold in the number of interviews to be conducted over a specific period.

This finding is also of use for adapting the frequency of DHQ controls to the number of interviews performed, HPs with a high number of interviews needing less frequent controls than those with a low number of interviews.

The variability between all of the HPs as a group and those who interviewed more than 500 donors during the study period encourages individual analysis of the data and the use of the analysis to set up specific strategies for each HP. Even if an HP is well-graded they can potentially improve their competency toward a better grade and continue to reduce the transfusion risk, even it will never be zero.

### Blood Donor Deferral Rate

Analysis of deferral rates is another efficient way of evaluating HP donor interviews and provides a different type of information than DHQ monitoring.

Deferral affects the supply of blood components because a blood bag is not collected and because the return rate for a further donation is reduced (19, 20), in particular, for first-time donors.

The comparison of individual deferral rates of an HP with the whole group allowed to identify discrepancies that were discussed with the monitored HP.

Examples:

- An HP had a much higher overall deferral rate than the group as a whole. Discussion with the supervisor revealed that this HP was very anxious about making mistakes, and preferred to defer donors if there was any doubt whatsoever.
- An HP had a much lower deferral rate for new sexual partners than the group as a whole. It appeared that this HP did not feel comfortable asking the question on this topic and relied entirely on the answer provided by the donor on the DHQ. Nevertheless, it is a well-known fact that quite a number of donors misunderstand or ignore the question and answer “no,” when in fact they should answer “yes.” Therefore, asking the question orally is important. Detection of this deviation was an opportunity to discuss this particular topic with the HP and provide additional training.

These two issues would not have been detected by means of DHQ monitoring; they illustrate the complementarity of the two blood donor selection evaluation tools used by the SFS.

These great inter-individual differences emphasize the importance of providing each HP with personal data in order to give individual advice and plan specific training.

It is important to take into account the various parameters, which may have an effect, such as the type of blood donation venue (Table 5). For instance, a frequent observation is that the number of deferrals due to sexual risk behavior is higher in blood collections organized in schools than in other types of venues, most probably due to the younger donor population (students). When an HP performs more donor selections in schools than average, it is logical for them to have a higher deferral rate than the HP group as a whole. On the other hand, the number of deferrals due to sexual risk behavior in blood collections organized in villages is below average. An HP who works mainly in village venues will, therefore, show a lower deferral rate for this risk factor. Donors’ deferral rates being statistically different among the types of donation venues, individual data have to be given to each HP with global data as a reference and with the distribution of their interviews in terms of donation venue. When these are considered, unexpected deviations can be selected and discussed with each individual HP, where applicable.

No hard data are available about the impact of the feedback provided by the supervisor to the HPs, but clear improvements were reported. An objective analysis of this impact may be valuable to validate the monitoring tools.

## Conclusion

In the SFS as in most blood transfusion centers, donor selection is the result of a pre-donation interview performed confidentially by an HP who has had full *ad hoc* training and has been qualified accordingly. The quality of the selection process is difficult to assess, but is important in the context of blood transfusion safety.

Having a supervisor present during the interview introduces a bias in itself, as both the donor and the HP may act differently compared to a plain face-to-face interview. Therefore, two complementary methods have been developed in the SFS to assess the quality of the selection process, i.e., *post hoc* control of the DHQs and analysis and comparison of deferral rates. Even if the number of DHQs checked was low compared to the total amount of donors selected, it made it possible to identify a number of mistakes made in donor selection, both at individual HP level as well as in a group of HPs. Deferral rates analyses also pinpointed the difficulties experienced by HPs in specific selection situations.

Providing the HPs with feedback on these findings is an opportunity to talk to them and draw their attention to some aspects of the selection process in order to improve it. In certain situations, the topic was included in a continued education session. Clear improvements were reported when further controls and/or deferral analyses were performed.

A comparison of the results for HPs according to the number of interviews conducted on an annual basis showed that for both the control of DHQs and the deferral rates, HPs with a greater number of interviews made fewer mistakes.

In conclusion, controls of DHQs and analyses of donor deferral rate seem to be useful tools to improve the quality of donor selection. It may be interesting to assess whether there is a threshold number of interviews, a HP should conduct per year in order to achieve optimal donor selection quality.

## Author Contributions

PG collected data recorded by HPs and performed a preliminary analysis of these data in order to develop figures and tables. EN and PG enhanced a deeper analysis of the data, and EN exploited the data with a view to planning individual and overall actions. EN and PG wrote the article.

## Conflict of Interest Statement

The authors declare that the research was conducted in the absence of any commercial or financial relationships that could be construed as a potential conflict of interest. The reviewer PT and the handling editor declared their shared affiliation.

## Abbreviations

DHQ, Donor Health Questionnaire; HP, health professional; QCF, Questionnaire Control Form; SFS, Service du Sang, Belgian Red Cross, French and German-speaking part of Belgium; TTI, transfusion-transmitted infections.
